# A Dental School in Atlanta

**Published:** 1887-06

**Authors:** 


### A DENTAL SCHOOL IN ATLANTA.

The recent addition of a Dental Department to the Southern Medical College is an important fact, and will give enlarged advantages and increased attractions to Atlanta as an educational center. It may be seen by reference to an advertisement in this issue of our journal that the facilities offered to dental students will be of a high order, while the charges are very moderate. Southern students, especially, should come to Atlanta as in all respects equal if not superior in its inducements to any other point for obtaining a dental education, and students from the North will find here a great advantage in the mildness of its climate where they may pursue their studies with greater comfort to themselves. Those who may have weak lungs or throat affections may come with much promise of relief to these troubles.

The same inducements hold with reference to students of medicine, who would do well to examine the claims presented in the Medical and Dental Departments of the Southern Medical College before deciding to go elsewhere.

The Chairs in the Dental Department have been ably filled by the election of the following gentlemen:

L. D. Carpenter, D.D.S., Prof. of Pathology and Therapeutics.

Wm. Crenshaw, D.D.S., Prof. of Operative and Clinical Dentistry.

J. S. Thompson, D.D.S., Prof. of Mechanical and Prosthetic Dentistry.

R. Y. Henley, D.D.S., Prof. of Oral Surgery and Dental Materia Medica.

S. G. Holland, D.D.S., Prof. of Chemistry and Metallurgy.

R. C. Word, M.D., Prof. of Physiology.

W. P. Nicolson, M.D , Prof. of Anatomy.

